# Survival after lobectomy versus sub-lobar resection in elderly with stage I NSCLC: a meta-analysis

**DOI:** 10.1186/s12893-019-0500-1

**Published:** 2019-04-15

**Authors:** Siyuan Dong, Steven A. Roberts, Shuang Chen, Xinwen Zhong, Shize Yang, Xiaohan Qu, Shun Xu

**Affiliations:** 1 Department of Thoracic Surgery, First Hospital of China Medical University, 155 North Nanjing Street, Shenyang, Liaoning China; 2 Ott lab, Massachusetts General Hospital, Harvard Medical School, 185 Cambridge Street, Boston, USA; 3 Department of Cardiovascular, First Hospital of China Medical University, 155 North Nanjing Street, Shenyang, Liaoning China

**Keywords:** Lobectomy, Sub-lobar resection, Elderly, Stage I NSCLC

## Abstract

**Background:**

We present a critical comparison of lobectomy and sub-lobar resection in elderly patients with early stage non-small cell lung cancer using meta-analytical techniques.

**Methods:**

A literature search was conducted between the period of December 1997 to March 2019 to identify the comparative studies evaluating 1-, 3-, and 5-year survival rates. The pooled odds ratios (OR) and the 95% confidence intervals (95% CI) were calculated with either the fixed or random effect models, respectively.

**Results:**

Six retrospective studies are included in our meta-analysis for a total of 1205 patients. 843 of the individuals were treated with lobectomy, while 362 were treated with sub-lobar resection. We found no significant difference between the lobectomy and the sub-lobar resection in either of the 1-, 3-, or 5-year survival rates.

**Conclusions:**

This study suggests that in elderly individuals with stage I NSCLC, a sub-lobar resection is statistically equivalent to the lobectomy in terms of 1-, 3-, and 5-year survival rates. Further large-scale randomized studies are needed to confirm our results.

## Background

Non-small cell lung cancer (NSCLC) is a leading cause of cancer-related deaths in individuals over 70 years old [1, 2], and its occurrence is likely to mirror the steady expansion of the elderly population.

Typically, treatment for localized stage I NSCLC consists of a lobectomy coupled with a systematic mediastinal lymphadenectomy [3]. While this treatment is generally accepted, recent reports have raised questions regarding the long term benefits of the lobectomy [4–6]. Alternatively, sub-lobar resection may provide an equivalent effect without many of the complications associated with lobectomy. To this end, Cancer and Leukemia Group B (CALGB) 140,503 [7] was established. This large, ongoing, multicenter randomized trial is currently evaluating whether sub-lobar resection is equivalent to lobectomy for the therapy of stage IA NSCLC 2 cm in diameter.

Due to the specific characteristics and limited sample sizes of the geriatric population, the elderly are significantly underrepresented in clinical research [8]. To date, very little has been done to compare the two procedures. Aside from complications that may occur during surgery, the elderly are much more susceptible to operative mortality and postoperative respiratory complications, especially if underweight [9]. Both the risks and benefits of surgical treatment should be assessed in the treatment of these patients. To determine whether lobectomy improves survival compared with sub-lobar resection in elderly with stage I NSCLC, we conducted a meta-analysis of elderly with stage I NSCLC who underwent lobectomy or sub-lobar resection. We evaluated the short-term and long-term survival rates of these individuals to achieve a more objective appraisal of the published research and provide a more precise comparison between the two surgical approaches.

## Methods

### Search strategy

Databases searched included the MEDLINE, Ovid MEDILINE, Cochrane Controlled Trial Register, Web of Science, PubMed and Embase databases in the period between December 1997 and March 2019. We included original research, reviews, meeting abstracts, editorials and letters as relevant sources of data. Search terms included, but not limited to: “lobectomy” “sub-lobar resection” “segmentectomy” “wedge resection” “elderly” “stage I” “non-small cell lung cancer” and “comparative study.” Such as in the PubMed the search strategy is: “lobectomy”[MeSH] or “lobectomy”[tiab] or “sub-lobar resection”[tiab] or “segmentectomy”[MeSH] or “segmentectomy”[tiab] or “wedge resection” [tiab] or “elderly”[tiab] or “stage I”[tiab] or “non-small cell lung cancer”[MeSH] or “non-small cell lung cancer”[tiab] or NSCLC [MeSH] or NSCLC [tiab]. In cases where full texts weren’t available, we contacted the corresponding authors to receive copies. Three authors (Shuang, Xinwen and Steven) independently searched the databases, while three authors (Shize, Shuang and Xinwen) independently reviewed 455 abstracts and 99 full texts independently. The Science Citation Index was used to cross-reference for further studies that meet the described criteria [10, 11].

### Study selection

Studies were included in our meta-analysis if (1) they included a comparison of the efficacy of lobectomy to that of sub-lobar resection in elderly with stage I NSCLC (where the elderly here is defined as ≥70 years); (2) they included a follow up ≥12 months following the procedure, and (3) the patients’ basic characteristics must be the same.

### Exclusion criteria

We excluded studies if (1) the research did not include a comparative group with treatment as a method of intervention; (2) subjects were treated for stage II or III NSCLC; (3) the focused on patients undergoing surgery for metastatic lung tumor; (4) there was an overlap between patient cohorts, centers, or authors evaluated in the published studies(here only the latest results were included); (5) the study was published more than two decades ago.

### Data extraction and quality assessment

The Downs and Black quality assessment method was used to evaluate the included articles [12]. This valuation includes 27 scoring standards, such as the clarity of the research objective, outcomes and characteristics of the patients, etc. Discrepancies between the three authors were solved by discussion among our team members and consensus with senior investigators (Siyuan and Shun). The final results were confirmed by two senior researchers (Xinwen and Siyuan).

### Statistical analyses

The Rev. Man 5.3.0. software package was used to conduct the meta-analysis. Odds ratio (OR) was calculated for the continuous outcomes while the mean difference with 95% confidence intervals (95% CI) was calculated for the dichotomous outcomes, respectively. A random-effects model and a fixed-effect model were used using“intention-to-treat” analysis. If results were not different between the two models, the random-effects model was reported, since this model was also used for the indirect comparisons. A significant difference between the two approaches was considered if the *P* value< 0.05. The I^2^ statistic was used to investigate the heterogeneity among the included trials [13]. The heterogeneity was explored by I^2^ and X^2^; I^2^ < 25% and I^2^ > 50% reflect small and large inconsistencies, respectively. *P* value < 0.05 were considered significant.

### Publication bias

Publication bias was assessed by constructing a funnel plot. The risk of bias of our research was evaluated by the asymmetry in the funnel plot of study size against curative efficacy.

## Results

### Description of the studies

**Fig. 1:**
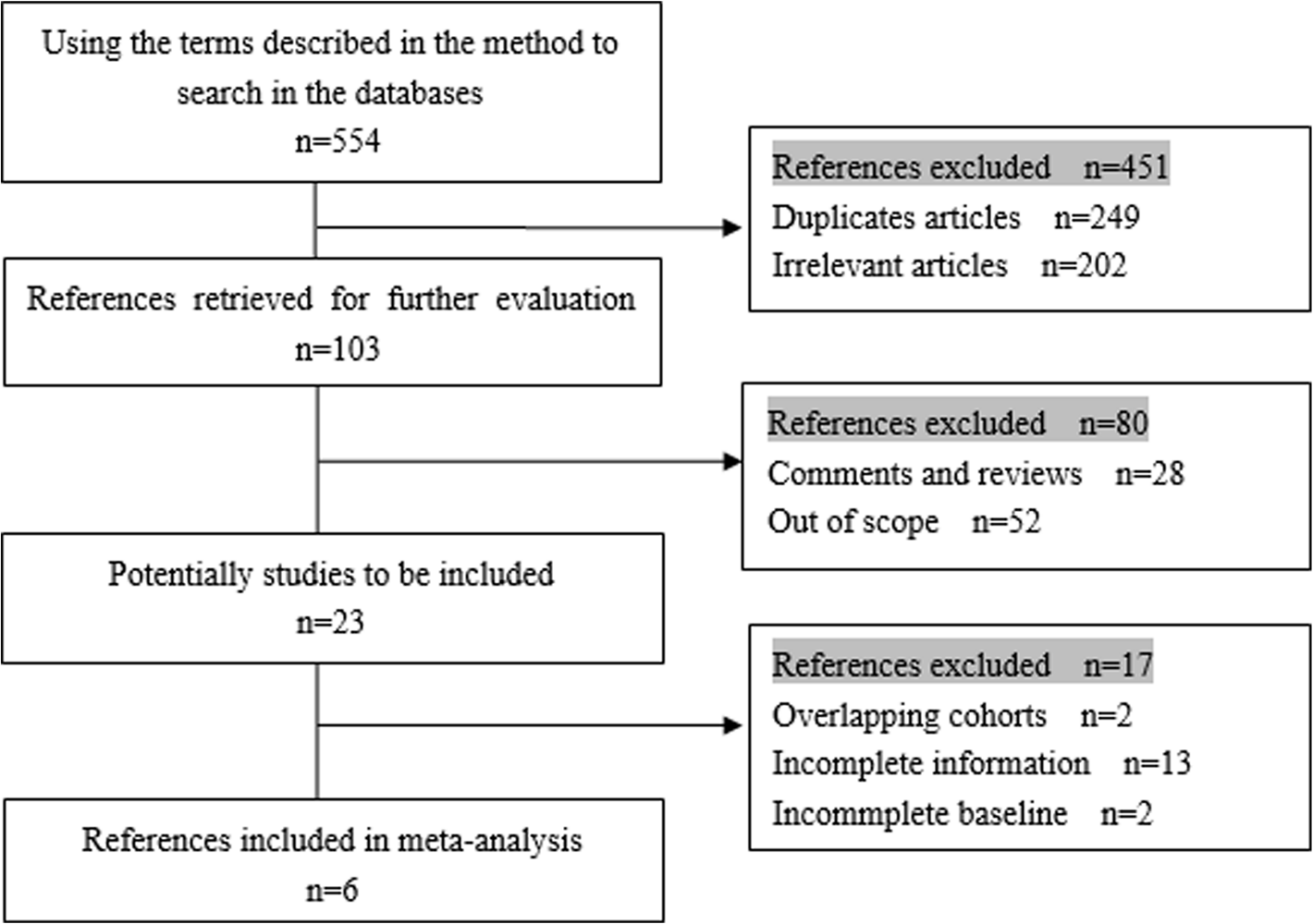
Flow chart illustrating papers selected for analysis

Six retrospective cohort studies that met our predetermined criteria were included in the dataset. A total of 1205 elderly individuals were included in the six articles; 843 patients were distributed to the lobectomy group, and 362 patients were distributed to the sub-lobar resection group to evaluate their 1, 3 and 5-year survival rate. The search procedure, results of the search strategies and selection criteria are shown in Fig. 1. The evaluation index and patient’s characteristics are summarized in Table 1.

**Table 1:** The characteristics and evaluation index of the included studies

Study	Year	Design	Country	Mean age(L/S)	Gender(M/F)	NO(L/S)	NO(W/Se)	5-y Survival %(L/S)	Assessment Score
Okami [26]	2010	OC	Japan	77/78	84/59	79/54	21/33	74.3/67.6	20
Richard [20]	2013	OC	UK	72/70	NR	375/158	158/0	58.7/55.1	18
Zhang [42]	2012	OC	China	83.6	39/13	32/20	16/4	21.5/18.7	17
Lin [41]	2013	OC	China	73/74	29/18	33/14	14/0	NR	19
Andrea [30]	2013	OC	Italy	78.1	257/62	202/71	NR	40.0/38.0	15
Liu [4]	2014	OC	China	≥70	96/71	122/45	NR	63.4/60.9	14

*OC* Observational cohort, *L* Lobectomy, *S* Sub-Lobar Resection, *NR* Not reported, *W* Wedge resection, *Se* Segmentectomy, *M* Male, *F* Female

### Evaluation of short and long term survival rate

**Fig. 2:**
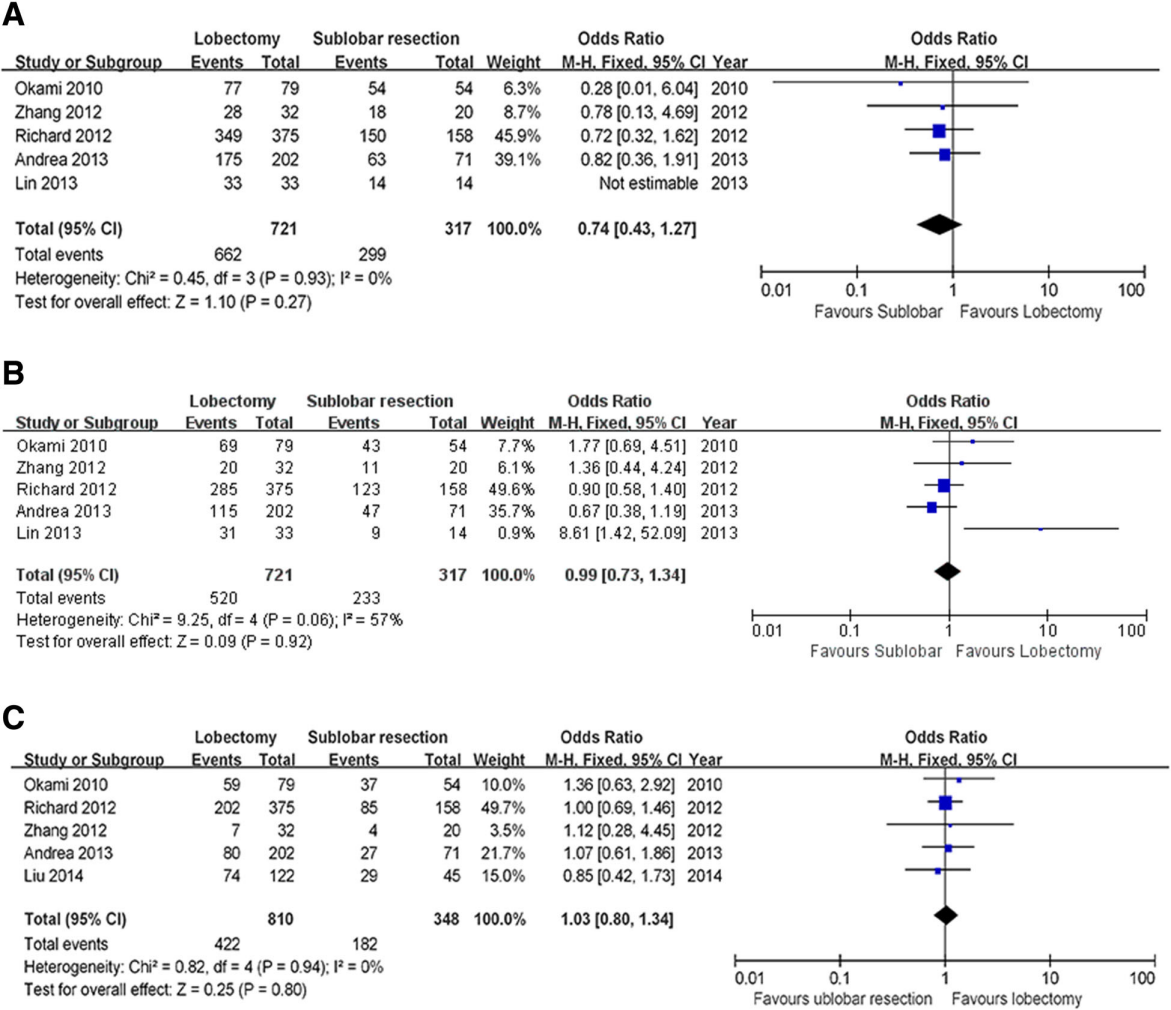
1(**a**), 3 (**b**) and 5(**c**)-year survival rate. Figure 2 1(**a**), 3(**b**) and 5(**c**)-year survival rate Forest plot of the Odds Ratio (OR) of the 1, 3 and 5-year survival rate following lobectomy versus sub-lobar resection for elderly with stage I NSCLC

Five of the six studies reported the one-year survival result. We found no significant difference among these studies when evaluated using a fixed effect model (X^2^ = 0.45, *P* = 0.93, I^2^ = 0%). The final combined outcome is shown in Fig. 2a (OR = 0.74; 95% CI 0.43–1.27; *P* = 0.27). In addition, five of the six studies reported three-year survival outcomes. We evaluated the heterogeneity of these results, again using the fixed effect model (X^2^ = 9.25, *P* = 0.06, I^2^ = 57%); these results are depicted in (Fig. 2b) (OR = 0.99; 95%CI 0.73–1.34; *P* = 0.92). Finally, five of these six included studies included the five-year survival outcome (OR = 1.03; 95%CI, 0.80–1.34; *P* = 0.80. Unlike the one- and three-year survival data, we found a statistically significant difference among these results(x^2^ = 0.82, *P* = 0.94, I^2^ = 0%) (Fig. 2c). All of the outcomes were summarized in Table 2.

**Table 2:** Summary of the results between lobectomy and sub-lobar of elderly with stage I NSCLC

Variables	Results		OR	*P*-value	I^2^
	lobectomy	sub-lobar			
1-y survival	91%	94%	0.74	0.27	0%
3-y survival	72%	74%	0.99	0.92	57%
5-y survival	52%	52%	1.03	0.80	0%

### Publication bias

**Fig. 3:**
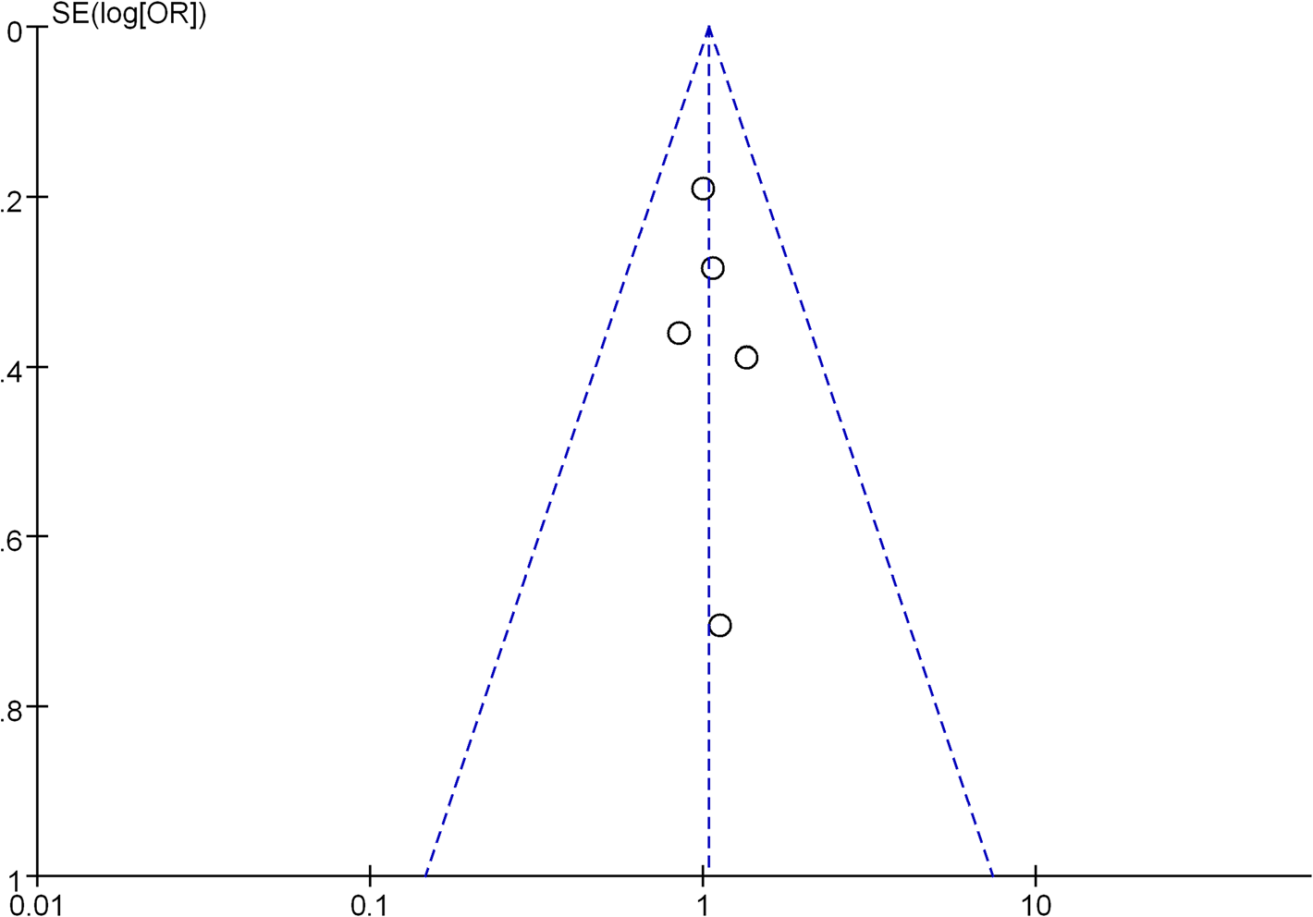
Funnel plot of the outcome of five-year survival rate

If non-significant results unpublished, there may be the publication bias occur. This has the effect of magnifying the apparent magnitude of the result artificially. The funnel plots of our meta-analysis are exhibited as Fig. 3. The funnel plot of the five-year survival receiving lobectomy and sub-lobar resection for the therapy of elderly with stage I non-small cell lung cancer manifested an asymmetry, revealing our research might contain publication bias.

## Discussion

With the widespread use of high resolution computed tomography, more than 80 % of pulmonary tumors are discovered at an early stage [14, 15]. The global aging population is increasing rapidly [16], and the pulmonary malignant is a disease of generally occurring in the elderly. Effective and efficient therapies are pressing needed to cope with the dramatic increase in the amount of early-stage NSCLCs [17]. At present, the widely accepted surgical approach for the treatment of NSCLC is lobectomy with systematic mediastinal lymphadenectomy. This common practice of treatment was confirmed by Lung Cancer Study Group (LCSG) 821 trial which revealed that lobectomy contribute fewer local recurrences and relative improved long-term survival [18]. In addition, recent study by Luzzi’s [19] et al. reported sub-lobar resection is followed with a significantly lower five-year survival compared with lobectomy (63% vs. 90%). However, these results may be obscured by comorbid illnesses such as COPD, diabetes and coronary artery disease, which are often present in elderly diagnosed with NSCLC [20, 21]. The choice between sub-lobar resection versus lobectomy is influenced by the surgeon’s and each institution’s experience and preference. More evidence is needed to guide clinical decision-making that balances both therapeutic efficacy and surgical risk in this special population.

Researchers who favor sub-lobar resection argue early stage NSCLCs are less aggressive than late stage [22, 23]. Analysis of the SEER database supports this conclusion; the survival outcomes for elderly patients with small peripheral tumors are comparable between lobectomy and sub-lobar resection [24]. These results have also been confirmed by the Japanese Joint Committee of Lung Cancer Registry [25]. In addition, research by other groups has demonstrated a high survival can be obtained by sub-lobar resection with a less extensive resection [5, 26–28]. A multivariate analyses by Brock et al. [29] showed poor respiratory function especially low FEV1, diminished physical status and tumor stage were associated with poor prognosis. As we know that the elderly are often associated with respiratory insufficiency [30]. Sub-lobar resections may provide an alternative of raising resection rates in aged patient with both NSCLC and poor respiratory function, so the elderly with significant deterioration of respiratory reserve can benefit from sub-lobar resection rather than traditional standard lobectomy [31, 32]. And the sub-lobar resection group also obtained a lower percentage of post-operative complications, especially the respiratory complications. What’s more, sub-lobar resection is accompanied with lower post-operative atelectasis, pneumonia and prolonged air leak rates than standard lobectomy group but without significant decreases in terms of in-hospital mortality [33].

In another aspect, researchers who emphasize that the lobectomy is helpful advocate that even the neoplasms which present localized by CT might already have micro-metastases. And there are researches that reveal sub-lobar resections are correlated with a significant increase of local recurrence [18, 34]. Study by EI-Sherif et al. conclude that, although sub-lobar resection result in a decreased rate of post-operative complications, it may also associated with lower disease-free survival [6]. What’s more, Dominguez-Ventura et al. reported that low forced expiratory volume in one second (FEV1) doesn’t impact the survival [35]. Rami-Porta suggested that limited resections shouldn’t be the first choice in patients who can accept standard lobectomy [36]. According to the European Respiratory Society/European Society of Thoracic Surgeons recommendations [37] and the American College of Chest Physician guidelines [38], for the reason that the heterogeneity in this specific population of aged patients, operation should not be denied only based on age. So other factors shouldn’t be neglected, such as the overall physical condition.

It’s remains a question of debate and inquiry that in the elderly whether sub-lobar resection is equivalent to standard lobectomy. To further test this hypothesis, all the candidates younger than 70-year were not included for the reason that these patients have both different baseline characteristics and life expectancies. This separates our current research from previous meta-analysis and randomized controlled trial in that this study examined only the subgroup of stage I NSCLC individuals that older than 70 years. This meta-analysis suggests that, for the elderly who are unsuitable to receive standard lobectomy, limited sub-lobar resection is an alternative which will get comparable benefits. We attribute this phenomenon to the following reasons: First of all, elderly with NSCLC are often afflicted with various comorbidities simultaneously, thus the perioperative management is more important and complex. The main reasons for equivalent oncological results in geriatric patients receiving limited sub-lobar resection compared to those who undergoing standard lobectomy are reduced postoperative complications, less surgical stress and better preservation of the pulmonary function [39]. Second, the geriatric patients are often associated with other age-related diseases and have a short life expectancy. As a result, they typically succumb of diseases other than lung cancer [4]. Lastly, NSCLC in the elderly may be less invasive than in younger individuals [39]. Although they did not receive the standard operation, they also could get the almost equivalent outcomes. During the study we evaluated the recurrence rate of the two approaches to further substantiate our conclusions. However, we found the data was too limited to adequately compare the two groups. Because unexpected lymph node metastases are found during the operation in not less than 10% of patients with an early lesion, so routine PET-scanning is suggested [40]. And during the operation, mediastinal lymph node sampling is also suggested.

This study is the first meta-analysis focusing the oncological outcome of sub-lobar resection for the treatment of elderly with early-stage NSCLC. In our work, we observed that sub-lobar resection allowed for surgical resection in elderly with stage I NSCLC without compromising oncological results. However, currently there are no large randomized trials can corroborate these finding, and in most cases, retrospective findings are limited to a single institution and small observational studies. Lobar versus sub-lobar resection remains to be a controversial topic. The thoracic community awaits the results from two ongoing randomized clinical trials from the US (CALBG 140503, clinicaltrials.gov number: NCT00499330) and from Japan (JCOG 0802, UMIN Clinical Trials Registry: UMIN000001272) to further elucidate the outcome after sub-lobar resections. And more prospective randomized studies comparing the two different surgical strategies are needed. The funnel plot was presented with annotation that it showed asymmetry and it might suggest the publication bias. As we know, the standard surgical therapy for stage I NSCLC is still the lobectomy, if the researches got the same conclusion in the elderly population, the researcher might abandon to publish the work. So our research might have publication bias.

However, there are several limitations in our research. No randomized controlled trials existing in this field to compare lobectomy with sub-lobar resection have been conducted. Most studies are limited to single institution case series and small observational studies. Because of this, there are only a total of 1205 elderly were included in the two groups. Two of all the included researches comprise almost 66% of all the elderly, and one study contains only 47 patients. Lobar versus sub-lobar resection remains to be a controversial topic and many studies have been published over the last 10 years. They all suffers the same problem that data did not take into consideration the differences in patient selection for sub-lobar resection. Was it because of age, comorbidity or poor lung function? Was it because of GGO? The defect of this article is that there are so many discrepancies among centers on their policy, procedures, and philosophy. Although we confined all the studies regarding stage I NSCLC. The subtypes of NSCLC were not classified in the included studies [41]. They may also affect the survival and recurrence. Additional randomized controlled trials in the studies we accessed would have increased the strength of our results. Last, there were no data available to distinguish between patients who did or did not receive adjuvant chemo-radiation therapy. Our research include six articles, publication bias evaluation using funnel plots is not ideal when total number of publications included in the pooled analysis are less than ten. So inability to assess publication bias accurately using funnel plots maybe exist in our research. There is also a bias for the English language.

## Conclusion

In our meta-analysis, we found that for elderly with stage I NSCLC, comparing lobectomy with sub-lobar resection showed almost equivalent survival rates. Further study is needed, and a large multicenter randomized trial comparing lobectomy with sub-lobar resection for the elderly with stage I NSCLC would be ideal.

## Abbreviations

95% CI: 95% confidence intervals
FEV1: Forced expiratory volume in one second
NSCLC: Non-small cell lung cancer
OR: Odds ratios
